# Supplementary material for: A bilateral interfacial passivation strategy promoting efficiency and stability of perovskite quantum dot light-emitting diodes
Source: Nat Commun. 2020 Aug 6;11:3902. doi: 10.1038/s41467-020-17633-3 (PMC7413529; doi:10.1038/s41467-020-17633-3)
Supplement: Supplementary file 1 — Supplementary Information [file 41467_2020_17633_MOESM1_ESM.pdf]

Supplementary Information

A Bilateral interfacial passivation strategy promoting efficiency and stability of perovskite quantum dot light-emitting diodes

Xu et al.

Supplementary Figures

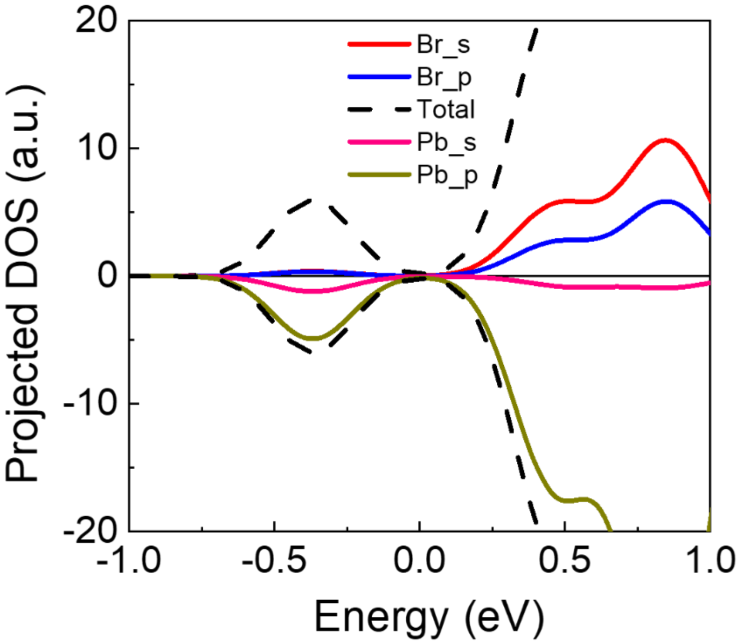

Supplementary Figure 1 In order to observe differect orbital contribution in trap state, we plot DOS of $CsPbBr_3$ with Br vacancy in the surface. We can find that both trap states and band dege are mainly contributed by Pb 6p orbitals in the unpassivated system. Source data are provided as a Source Data file.

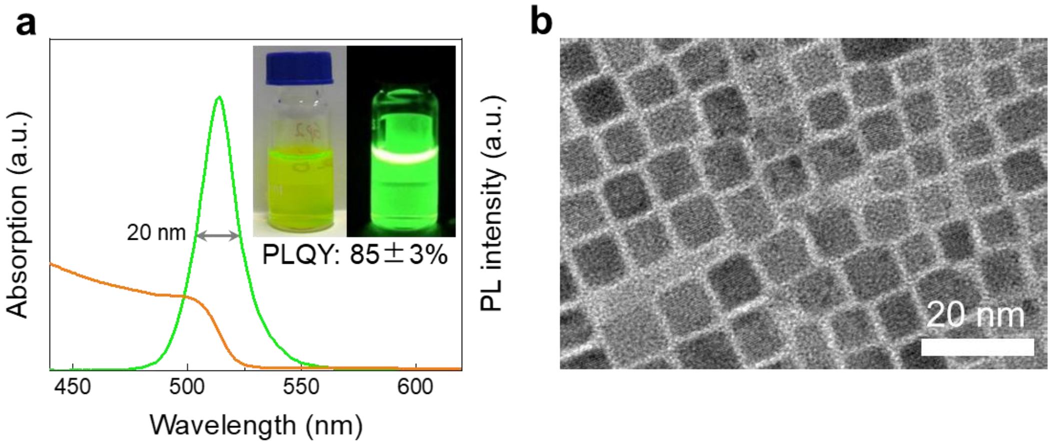

Supplementary Figure 2 The optical and microstructure of $CsPbBr_3$ QDs. (a) UV−vis absorption and PL spectrum of purified $CsPbBr_3$ QDs dissolved in 1-octane. Inset is the photograph of the purified $CsPbBr_3$ QD inks under natural light and UV light. (b) Transmission electron microscopy of $CsPbBr_3$ QDs. Source data are provided as a Source Data file.

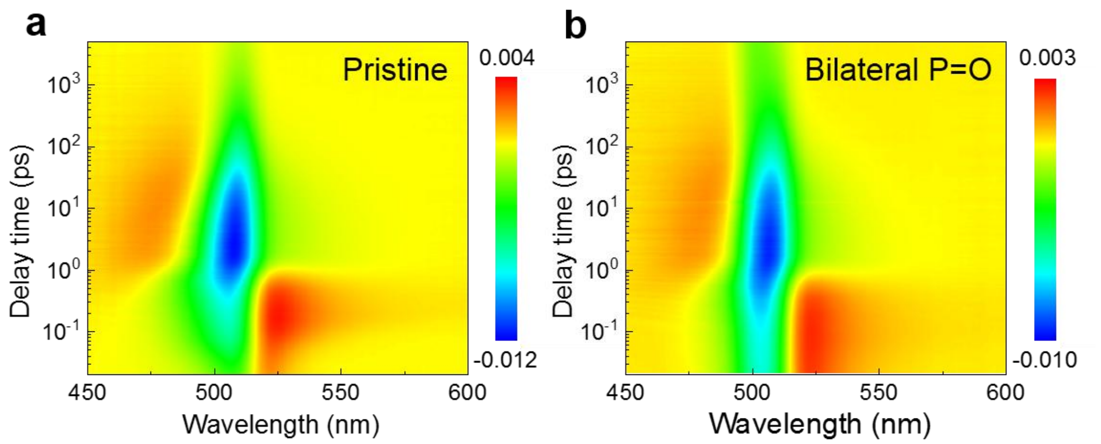

Supplementary Figure 3 (a) and (b) Typical TA spectra mappings of pristine and TSPO1-passivated QD films. Source data are provided as a Source Data file.

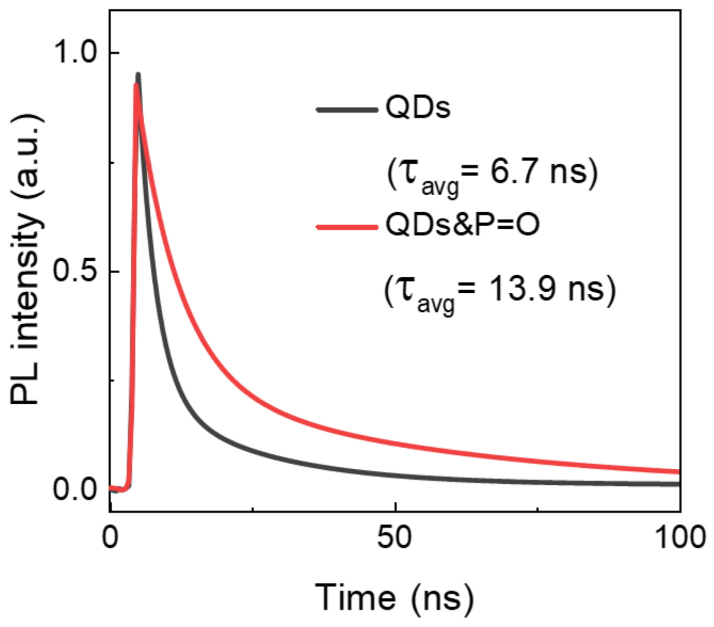

Supplementary Figure 4 PL decay curves of primal and TSPO1-passivated $CsPbBr_3$ QD films. Source data are provided as a Source Data file.

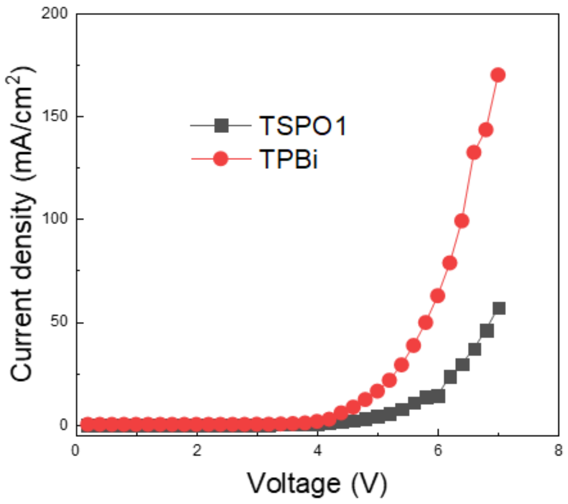

Supplementary Figure 5 Electrical properties demonstrated by current density vs. voltage curves of electro-only devices based on TSPO1 and TPBi. The structure of ITO/ZnO/TSPO1/Alq3/Al and ITO/ZnO/TPBi/Alq3/Al are designed for the comparison of the mobility. Source data are provided as a Source Data file.

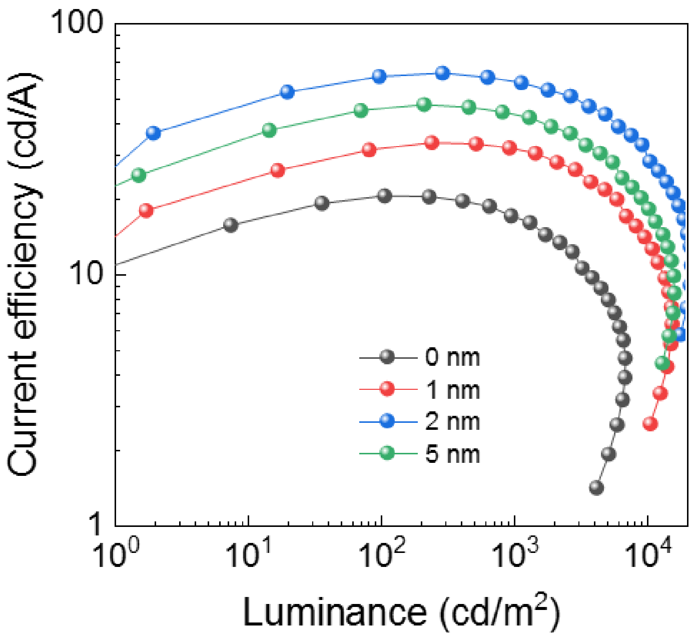

Supplementary Figure 6 Current efficiency of the unilateral devices (on top of QD film) as a function of luminance. Source data are provided as a Source Data file.

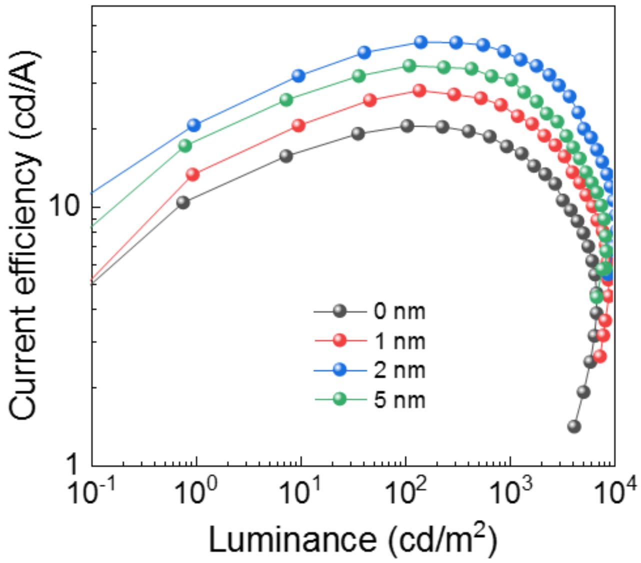

Supplementary Figure 7 Current efficiency of the unilateral devices (on bottom of QD film) as a function of luminance. Source data are provided as a Source Data file.

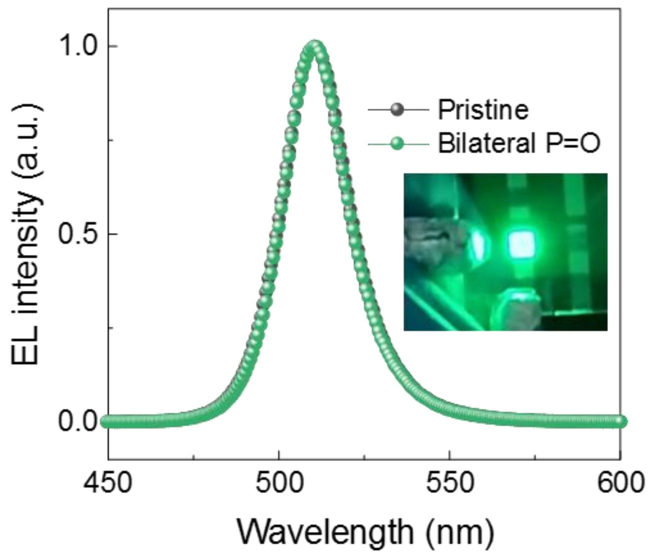

Supplementary Figure 8 Normalized EL spectra of pristine and bilateral-passivation QLEDs under applied voltage of 3 V. Source data are provided as a Source Data file.

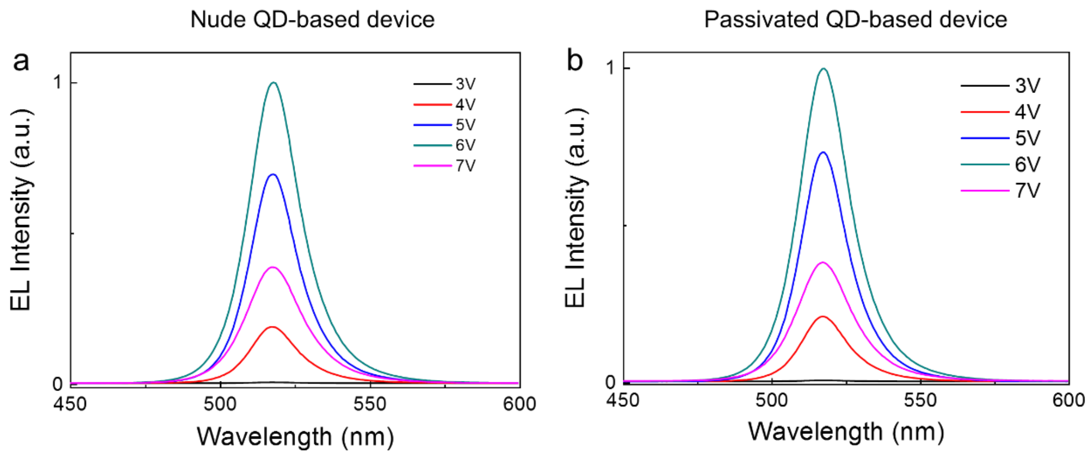

Supplementary Figure 9 The EL spectra of (a) nude QD-based device and (b) passivated QD based device under different driving voltage. Source data are provided as a Source Data file.

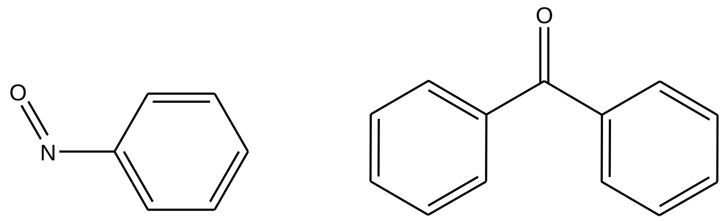

nitrosobenzene benzophenone

Supplementary Figure 10 The molecular structure of nitrosobenzene (~N=O) and benzophenone (~C=O). Source data are provided as a Source Data file.

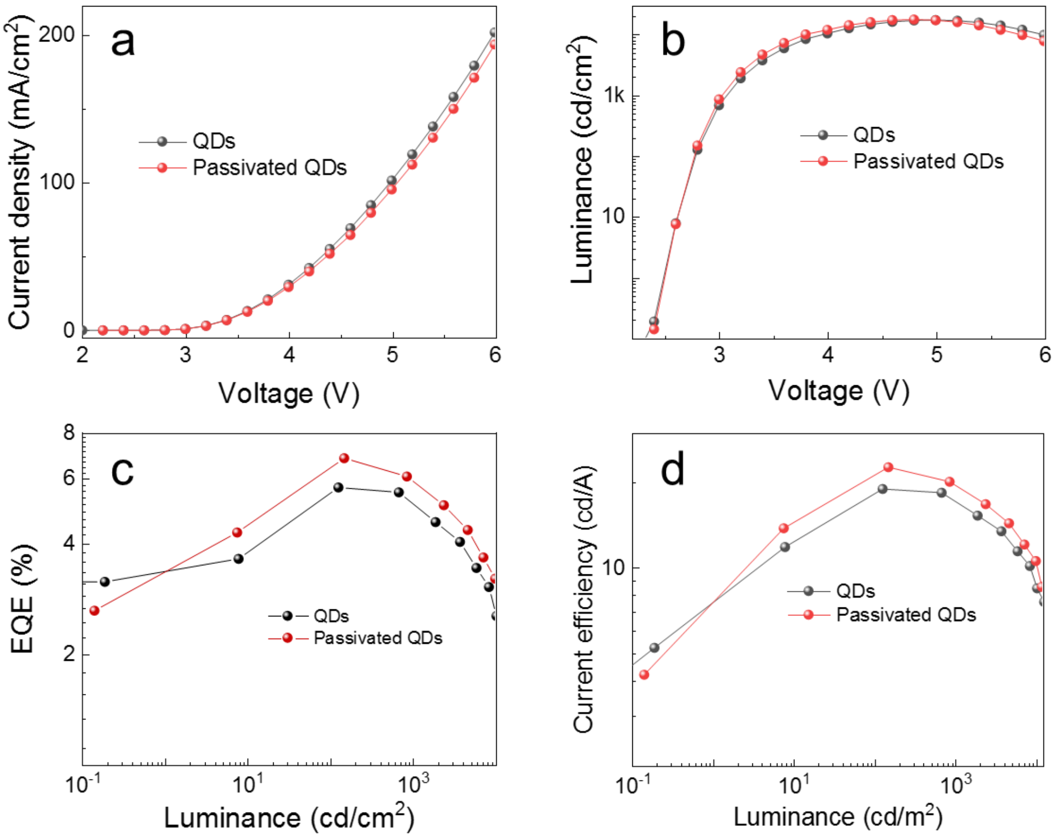

Supplementary Figure 11 EL performances of pure $CsPbBr_3$ QLED and bilateral-passivated QLED with benzophenone. Source data are provided as a Source Data file.

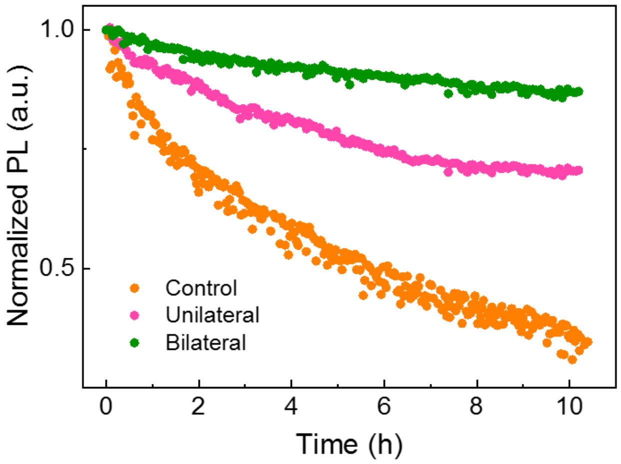

Supplementary Figure 12 The PL stability of pristine unilateral passivated and bilateral passivated QD films under continuous illumination (365 nm) in ambient air with RH 40%. Source data are provided as a Source Data file.

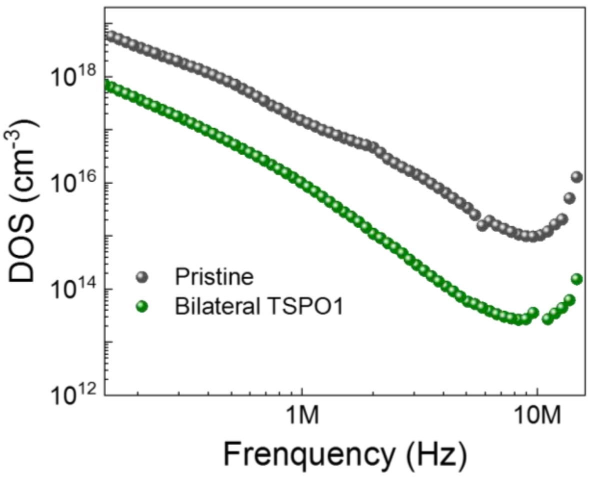

Supplementary Figure 13 Density of states of pristine perovskite device and bilateral-passivated device with TSPO1 from DLCP measurement. Source data are provided as a Source Data file.

Supplementary Notes

Supplementary Note 1. DOS in Supplementary Figure 13 is calculated according to following method. Capacitance with respect to amplitude was obtained to correct the capacitance value at higher orders, following the equation (1)

$$\mathrm{C} = C_0 + C_1 dV + C_2 (dV)^2 + \cdots \quad (1)$$

The density of states (DOS) for the devices with variation in frequency was calculated using the following equation (2):

$$\mathrm{N} = -\frac{C_0^3}{2q\varepsilon A^2 C_1} \quad (2)$$

C_0 and C_1 are obtained by quadratic polynomial fitting of equation 1, q is the elementary charge, ε is the relative dielectric constants of $CsPbBr_3$, A is the junction area.

Supplementary Methods

Fabrication of electron-only device: After the ITO-coated glass substrate was cleaned and sonicated, TPBi (40 nm) was deposited on the substrate using a thermal evaporation system through a shadow mask under a high vacuum of ~2×10^{-4} Pa, Then $CsPbBr_3$ QDs were deposited by spin coating at 2000 r.p.m. for 60 s, QD layers were baked at 60 ºC for 10 min. Finally, TPBi (40 nm) and LiF/Al electrodes (1 nm/100 nm) were deposited through thermal evaporation.

ITO/PEDOT:PSS/PTAA/QDs/TPD/Al

Fabrication of hole-only device: After the ITO-coated glass substrate was cleaned and sonicated, PEDOT:PSS solutions were spin-coated onto the ITO-coated glass substrates at 4000 r.p.m. for 60 s and baked at 140 ºC for 15 min. PTAA (in chlorobenzene 5 mg mL^{-1}) and $CsPbBr_3$ QDs (in n-octane 20 mg mL^{-1}) were deposited by layer-by-layer by spin coating at 2000 r.p.m. for 60 s. Finally, TPD (40 nm) and Al electrodes 100 nm) were deposited using a thermal evaporation system through a shadow mask under a high vacuum of ~2×10^{-4} Pa.
